# Combination therapy is it in the future for successfully treating peripheral diabetic neuropathy?

**DOI:** 10.3389/fendo.2024.1357859

**Published:** 2024-05-15

**Authors:** Mark Yorek

**Affiliations:** ^1^ Department of Internal Medicine, University of Iowa, Iowa City, IA, United States; ^2^ Department of Veterans Affairs Iowa City Health Care System, Iowa City, IA, United States; ^3^ Fraternal Order of Eagles Diabetes Research Center, University of Iowa, Iowa City, IA, United States

**Keywords:** diabetes, peripheral neuropathy, animal models, oxidative stress, inflammatory stress

## Abstract

In 2022, the Center for Disease Control and Prevention reported that 11.3% of the United States population, 37.3 million people, had diabetes and 38% of the population had prediabetes. A large American study conducted in 2021 and supported by many other studies, concluded that about 47% of diabetes patients have peripheral neuropathy and that diabetic neuropathy was present in 7.5% of patients at the time of diabetes diagnosis. In subjects deemed to be pre-diabetes and impaired glucose tolerance there was a wide range of prevalence estimates (interquartile range (IQR): 6%-34%), but most studies (72%) reported a prevalence of peripheral neuropathy ≥10%. There is no recognized treatment for diabetic peripheral neuropathy (DPN) other than good blood glucose control. Good glycemic control slows progression of DPN in patients with type 1 diabetes but for patients with type 2 diabetes it is less effective. With obesity and type 2 diabetes at epidemic levels the need of a treatment for DPN could not be more important. In this article I will first present background information on the “primary” mechanisms shown from pre-clinical studies to contribute to DPN and then discuss mono- and combination therapies that have demonstrated efficacy in animal studies and may have success when translated to human subjects. I like to compare the challenge of finding an effective treatment for DPN to the ongoing work being done to treat hypertension. Combination therapy is the recognized approach used to normalize blood pressure often requiring two, three or more drugs in addition to lifestyle modification to achieve the desired outcome. Hypertension, like DPN, is a progressive disease caused by multiple mechanisms. Therefore, it seems likely as well as logical that combination therapy combined with lifestyle adjustments will be required to successfully treat DPN.

## Diabetic peripheral neuropathy

About 50% of the diabetes population is affected by DPN. After many years of pre-clinical and clinical research a life changing treatment remains elusive. Animal studies have shown that the cause of DPN is complex with numerous mechanisms being involved at different cellular levels including vascular, nerve and Schwann cells. This complex etiology is believed to be the primary factors contributing to sensory and motor nerve impairment (1–5). Elevated and dysregulated blood glucose levels were thought to be the sole cause of peripheral neuropathy in patients with type 1 diabetes. In patients with type 1 diabetes good regulation of blood glucose levels and hemoglobin A_1_C values was shown to slow the progression of DPN. However, good glycemic control does not prevent, nor is it sufficient to significantly alter the course of DPN in patients with type 2 diabetes (1, 2, 5). To further complicate this issue research in human subjects has shown that about 30% of those with impaired glucose tolerance and insulin resistance develop forms of sensory neuropathy (6, 7). This observation has been duplicated in studies with diet-induced obese rats that develop sensory neuropathy related deficits (8).

## Etiology of DPN

In 2004 Dr. Brownlee presented the Banting lecture that focused on a unifying hypothesis for diabetes complications (9). In this presentation four pathways were highlighted, and these are briefly reviewed below. Unfortunately, the etiology of diabetes complications and DPN is not that straightforward. There are many other factors that contribute to these complications although ultimately increased oxidative stress and/or inflammation is a contributing factor and maybe the common element linking many of these other pathways to the pathology of DPN. Below I also briefly review the pathology of oxidative and inflammatory stress in DPN and why combination therapies may be the best approach to ultimately finding a treatment that will work.

## Aldose reductase pathway

**Figure 1:**
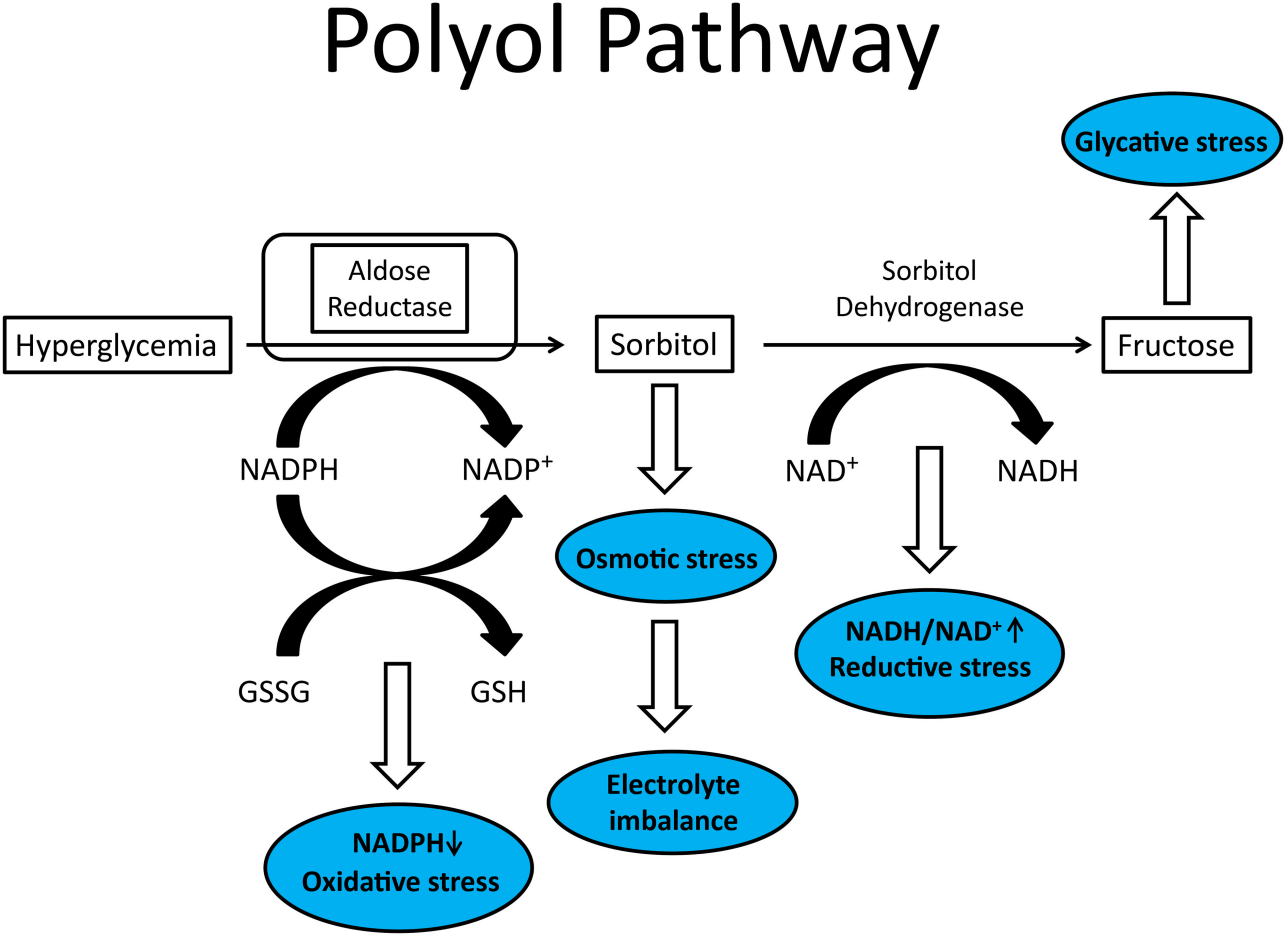
Illustration of polyol pathway with blue ovals representing conditions that contributes to diabetic peripheral neuropathy.

One of the early mechanisms to receive a great deal of attention was the aldose reductase pathway or polyol pathway. This pathway consists of two enzymes: aldose reductase and sorbitol dehydrogenase (10). In the initial step aldose reductase reduces glucose to sorbitol in a reaction requiring NADPH. This is followed by sorbitol reacting with NAD-dependent sorbitol dehydrogenase to form fructose (**Figure 1**). The increase in cellular sorbitol induces osmotic stress that is compensated at the cellular level by decreasing the levels of taurine and myo-inositol. Besides reduction of intracellular levels of myo-inositol and taurine other potential negative pathological consequences of this pathway are the generation of excess fructose, a potent glycating agent, and a decrease in nerve Na^+^/K^+^ ATPase activity.

Preclinical studies treating primarily diabetic rodents with aldose reductase inhibitors demonstrated improvement in multiple endpoints associated with DPN (11–13). This led investigators to propose what became known as the compatible osmolyte hypothesis: myo-inositol depletion and abnormal signaling by phosphatidylinositol causes a decrease in peripheral nerve Na^+^/K^+^ ATPase activity (14). Na^+^/K^+^ ATPase is important for maintaining the sodium and potassium transmembrane potential that is required for nerve impulse conduction as well as the sodium gradient used for uptake of substrates that are Na^+^-dependent (15). Treatment with aldose reductase inhibitors not only corrected the depletion of intracellular myo-inositol and taurine in tissues but sciatic nerve Na^+^-ATPase activity was also corrected as was DPN as determined by measurement of nerve conduction velocity. Supplementing diets with myo-inositol or taurine was also shown to partially reverse peripheral neuropathy in diabetic rodents (16–18). My laboratory has demonstrated that treating streptozotocin-induced type 1 diabetic rats with an aldose reductase inhibitor or myo-inositol dietary replenishment improved endoneurial blood flow and dilatory function of epineurial arterioles that provide blood flow to the sciatic nerve (19). This study was important, because we had previously demonstrated that decreased vascular relaxation to acetylcholine by epineurial arterioles preceded the slowing of nerve conduction velocity suggesting that vascular dysregulation is a contributing factor to DPN (20).

The effect of aldose reductase inhibitors has been studied in human subjects with DPN. As expected, the design of these studies varied including the number of subjects enrolled, duration of treatment, dose, and endpoints examined. Wide variation in study design often leads to mixed results. Some of these human studies with aldose reductase inhibitors reported significant improvement in nerve conduction velocity and axonal atrophy (21–23) while others reported non-significant results (24–26). The results from these trials were summarized nicely by Pfeifer et al. (27) stating that “future trials should be designed with adequate statistical power, with consideration of the variability of the endpoint measurements for long enough duration, and with rigorous quality control to definitively confirm the utility of aldose reductase inhibitors in the treatment of diabetic distal symmetrical polyneuropathy and autonomic neuropathy”. This review was written in 1997 and the same problems are still a challenge for adequate clinical trials for DPN in 2023. Presently, in Japan, Epalrestat is the only aldose reductase inhibitor being used clinically for treatment of DPN (28, 29). Despite these mixed results, aldose reductase is still considered to be a potential therapeutic target for neurodegeneration (30).

## Non-enzymatic glycation

**Figure 2:**
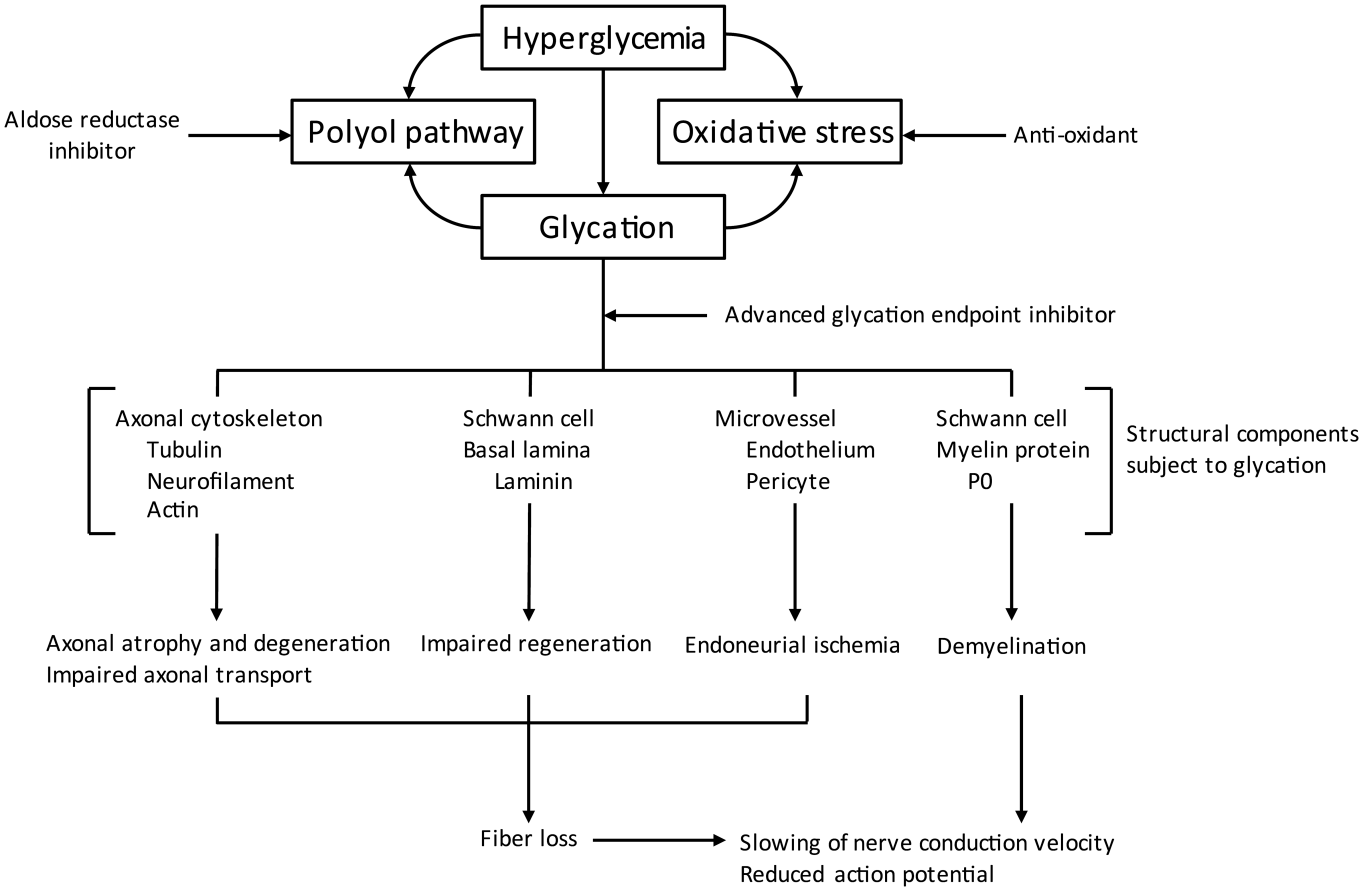
Pathogenic mechanisms associated with non-enzymatic glycation (modified from (31).

Along with the polyol pathway, increased non-enzymatic glycation was the other pathway that was recognized early to contribute to DPN. When present in over abundant levels reducing sugars like glucose or fructose react non-enzymatically with free amino groups of proteins, lipids, or nucleic acids to initially form Schiff bases or Amadori adducts (**Figure 2**) (31–33). Left unchallenged these products will continue to undergo reactions that lead to the formation of advanced glycation endproducts (32). In diabetes, this accumulation can cause structural and functional damage to tissues and organs including components of peripheral nerves (34, 35). Besides their effect on nerves, advanced glycation endproducts can alter vascular structure and function, reducing blood flow, and leading to ischemia. Pathology attributed to advanced glycation endproducts can also occur through binding of these compounds to the receptor for advanced glycation endproducts (RAGE) that is expressed in Schwann and endothelial cells (36, 37). Activation of RAGE has been demonstrated to increase expression of markers associated with increased oxidative stress (38, 39). In regard to DPN, treatment with inhibitors of advanced glycation end-product formation has been shown to improve endoneurial blood flow as well as other DPN related endpoints; thereby further linking vascular and neural complications to DPN (19, 40). In mice lacking RAGE the induction of type 1 diabetes by streptozotocin did not reduce nerve conduction velocity as compared to type 1 diabetic wild type mice (41, 42). Treating diabetic wild type mice with soluble RAGE (sRAGE), a treatment intended to sequester RAGE ligands prior to their binding to RAGE also improved DPN (42). An early inhibitor of formation and activity of advanced glycation endproducts was aminoguanidine (43). It has been widely shown that treating diabetic rats with aminoguanidine improved DPN (19, 43–48).

## Protein kinase C pathway

Diabetes induced elevation in circulating glucose levels leads to the accumulation of glucose in cells independent of insulin for glucose transport. Once transported into these cells, there is an increase in glycolysis and concentrations of diacylglycerol are elevated, which can activate PKC (49). Activity of PKC in neural tissue including the vasculature in diabetes is complex. When PKC is activated by intracellular hyperglycemia the expression of genes that target a variety of cellular systems is altered. For example, in vascular tissue diabetes increases the activity of PKCβ and endothelial nitric oxide synthase is decreased thereby reducing production of nitric oxide a vasodilator and the production of the vasoconstrictor entothelin-1 is increased (50). It is likely that PKCβ activation induces DPN through reduction of vascular blood flow and impairment of microvascular tissue rather than directly injuring neurons (50). Several laboratories have reported that inhibition of PKCβ improves vascular and neural function in diabetic rodents (51–54). In addition, dysregulation of Schwann cells in part through altered PKC activity can contribute to DPN (50, 55–58). In streptozotocin-induced diabetic rats’ activity of PKCα was decreased in neurons and Schwann cells that was corrected by treatment with methylcobalamin (59). In contrast, up regulation of PKCϵ in DPN has been associated with endoplasmic reticulum stress, autophagic formation and pain (60).

## Hexosamine pathway

Cellular hyperglycemia is also critical to the pathology of this pathway as is in the polyol pathway and excess production of diacylglycerol for activation of PKC. When excess glucose is being metabolized by the glycolytic pathway an excess amount of the fructose-6-phosphate that is formed gets diverted into the hexosamine pathway leading to the over production of uridine diphosphate *N*-acetyl glucosamine. This excess glucosamine gets incorporated onto the serine and threonine residues of transcription factors such as Sp1 resulting in pathologic changes in gene expression of fibrinogen activator inhibitor-1 and transforming growth factor-β (61). Treatment of rats with benfotiamine, a synthetic analogue of thiamine (vitamin B1) and an inhibitor of hexosamine pathway and formation of advanced glycation end products, has been shown to improve DPN (62). In human subjects with DPN it has also been shown to be beneficial, however; it is not commonly used and less effective than α-lipoic acid (63–65).

## Oxidative stress

In the Banting lecture Brownlee referred to overproduction of superoxide (O2^-^) by the mitochondrial electron transport chain as the one element linking the different pathogenic pathways to diabetic complications (9).

Oxidative stress occurs is a result of an inability of cellular antioxidant mechanisms to neutralize increased generation of reactive oxygen species (ROS) (66). The most common forms of ROS are O_2_^-^, hydroxyl radical (OH^-^), hydrogen peroxide (H_2_O_2_), and peroxynitrite (ONOO^-^) (67). These compounds can be generated throughout the body under both normal and pathological conditions (67). O_2_^-^, the most biologically active form of ROS, is created via multiple sources including the electron transport chain, and various enzymes: NADH oxidase, NAD(P)H oxidase, lipoxygenase, cyclooxygenase, xanthine oxidase, cytochrome P-450, and, during periods of tetrahydrobiopterin deficiency, by nitric oxide synthase (67). O_2_^-^ can react with superoxide dismutase (SOD) acquiring an electron it is converted to H_2_O_2_. SOD consists of a family of enzymes: mitochondrial derived Mn-SOD and two isoforms of Cu,Zn-SOD, which are located either in the cytosol or extracellularly (67). H_2_O_2_ is converted to water by the action of catalase or glutathione peroxidase in the presence of reduced glutathione (67). In the presence of iron, H_2_O_2_ can form OH^-^ via a process known as the Fenton reaction (67). ONOO^-^ formation is the result of a reaction between O2^-^ and nitric oxide (NO). Referred to as nitrosative stress it causes damage primarily to vascular tissue and has been demonstrated to be increased with diabetes (67–69). ONOO^-^ biologically has a short half-life but has the ability to diffuse across cell membranes and depending on the cell environment can cause a nitrosylation of proteins and peroxidation of lipids (69).

As previously discussed, endothelial dysfunction occurs early in diabetes and is a primary factor in the development of diabetic vascular disease, which contributes to the development of DPN. Studies from numerous laboratories including my own have demonstrated that reducing oxidative stress in a diabetes setting improves vascular function and blood flow. Combined, these studies have demonstrated that the generation of ROS in diabetes such as O2^-^ and ONOO^-^ contributes to oxidative stress and vascular dysfunction including decreased endothelium-dependent vascular relaxation of epineurial arterioles of the sciatic nerve (19, 20, 70, 71).

**Figure 3:**
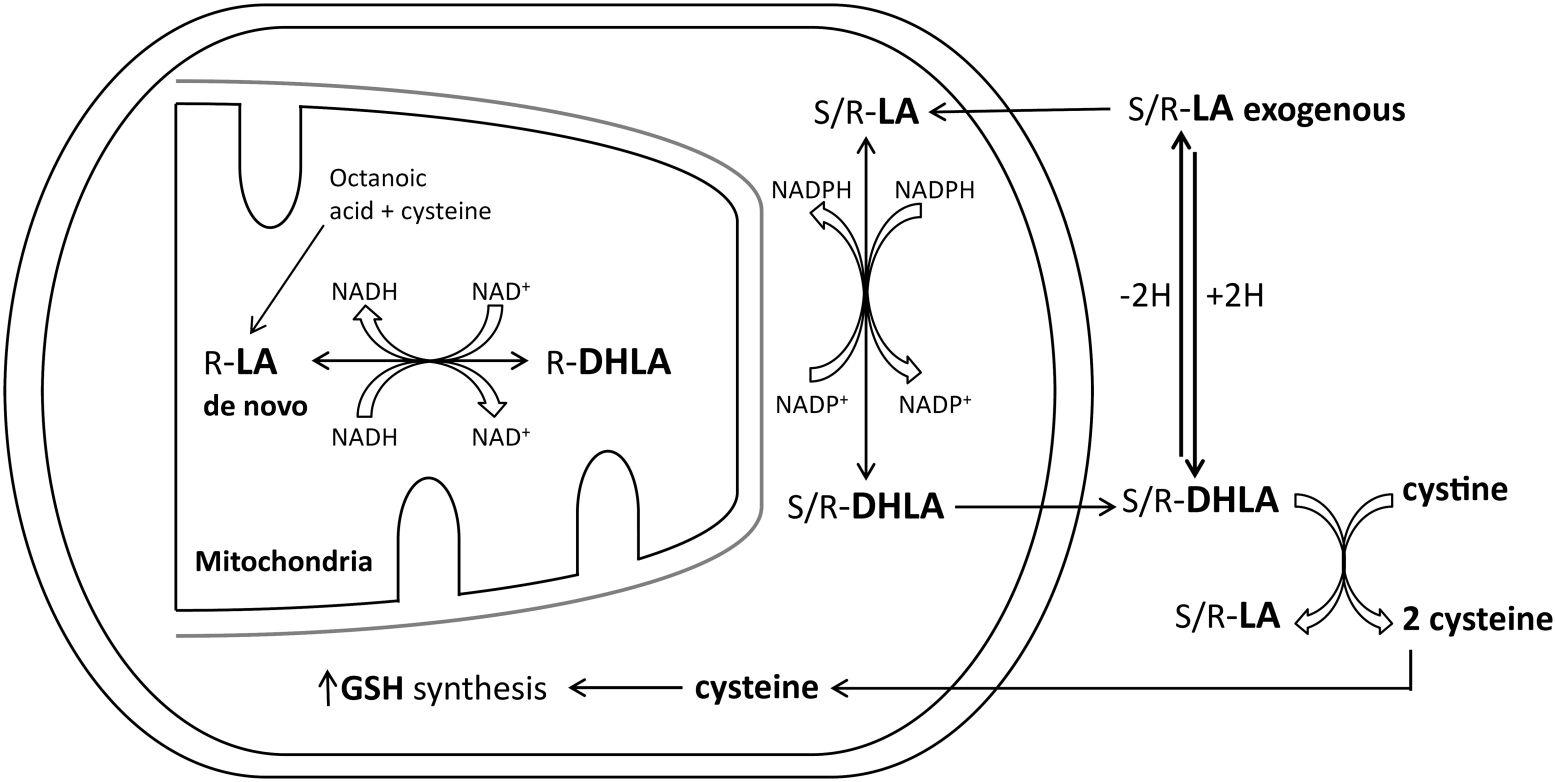
Lipoic acid (LA) and dihydrolipoic acid (DHLA) metabolism (modified from (77).

The most often studied antioxidant in a diabetes setting and DPN has been α-lipoic acid (72, 73). Using diabetic rats, we have observed that α-lipoic acid provides good protection against oxidative stress (70). We observed improvement in diabetes-induced decrease in endoneurial blood flow, endothelium-dependent vascular relaxation in arterioles that provide circulation to the sciatic nerve to acetylcholine, and nerve conduction velocity. α-Lipoic acid also reduced the production of O_2_^-^ by the aorta and O_2_^-^ and ONOO^-^ by epineurial arterioles. α-Lipoic acid is a good metal chelator and is capable of scavenging hydroxyl radicals, hypochlorous acid and singlet oxygen, but not O_2_^-^ or peroxyl radicals (73–76). However, in its reduced form, as dihydrolipoic acid, it is a good scavenger of O_2_^-^ and prevents initiation of lipid peroxidation (73–76). *In vivo*, α-lipoic acid can be converted into dihydrolipoic acid (73, 74) (**Figure 3**). This reaction requires NADPH; however, levels of this co-factor is reduced in diabetes due to the increased flux of glucose through the polyol pathway and the formation of sorbitol as discussed above (66, 78). To overcome this pathological condition a combination therapy using an aldose reductase inhibitor to protect the availability of NADPH with α-lipoic acid should provide improved treatment for DPN compared to monotherapy. In theory this combination will promote the formation of dihydrolipoic acid, thereby enhancing the antioxidant potential of α-lipoic acid and possibly providing a synergistic effect. To test this hypothesis my laboratory examined the effect of the combination of α-lipoic acid and fidarestat, an aldose reductase inhibitor, on vascular and neural complications in a type 1 diabetic rat model (79). We demonstrated that this combination was more efficacious in preventing diabetes-induced vascular and neural dysfunction than the use of α-lipoic acid and fidarestat alone. We attributed this outcome to the verified increased conversion of α-lipoic acid to the more efficient antioxidant dihydrolipoic acid (**Figure 3**) (77, 79). This approach has also been applied clinically. Two different teams in China compared the efficacy of epalrestat and α-lipoic acid vs. monotherapy Both teams reported that the combination of α-lipoic acid and epalrestat is better than monotherapy clinically for DPN and improvement of motor and sensory nerve conduction velocity (80–82).

There have been other combination therapies tested using α-lipoic acid. In a pre-clinical study using type 1 diabetic rats the combination of coenzyme Q10 with α-lipoic acid was found to reduce oxidative stress and prevent apoptosis and degeneration of dorsal root ganglion neurons (83). In a study using the combination of prostaglandin E1 and α-lipoic acid the investigators found that the combination therapy was superior to monotherapy in improving neuropathic symptoms and nerve conduction velocity in patients with DPN (84). Another team studying type 1 diabetic rats used the combination of a prostaglandin E1 analogue and the aldose reductase inhibitor, statil, improved nerve conduction velocity better than monotherapy (85). This team concluded that a multiple-drug therapy with different mechanisms of action has greater effects on DPN than a single-drug therapy and is worthy of clinical consideration (85).

## Inflammatory stress

A condition that is almost inseparable from oxidative stress is inflammatory stress. Inflammatory stress is defined as an overactivation of the immune system, causing an imbalance between accumulation of inflammatory molecules and ability anti-inflammation systems to neutralize their abundance. Chronic inflammation is an essential component of all chronic diseases including diabetes. Hyperglycemia coupled with loss of insulin signaling (type 1 diabetes) and/or insulin resistance (type 2 diabetes) including dysregulation of lipid metabolism and dyslipidemia, lead to systemic inflammation and vicious cycles of oxidative/nitrosative stress, endoplasmic and mitochondrial stress, and accumulating cellular damage (86). Besides nerve bodies, Schwann cells and microvasculature are also impaired by oxidative and inflammatory stress. Thus, an effective treatment for diabetes complications including DPN should include some form of an anti-inflammatory component.

## Mechanism related therapies

Pre-clinical studies have provided a wide array of potential mechanisms and effective treatments for DPN (50, 61, 87). However, when tested in human subjects there was a general failure although α-lipoic acid and epalrestat are used clinically for DPN in Germany and Japan, respectively. These treatments are not recommended universally by any guidelines (88). Why success in pre-clinical studies for DPN primarily in rodents did not translate to human subjects has been a highly debated subject. Obvious responses include that DPN in rodents does replicate human DPN well enough for translation. This is likely true for some DPN endpoints used for studies with diabetic rodents such as nerve conduction velocities. Slowing of nerve conduction velocity develops within weeks in diabetic rats while in humans it takes years to manifest clinically (20, 89). However, pre-clinical endpoints examining decrease in sensory nerve density in the skin and cornea of diabetic rodents does seem to correspond well to the human condition (8, 90). Other reasons for the failure of translating pre-clinical finding to human disease are the endpoints used in clinical studies for DPN are not sensitive or require greater period for reversal then is allowed for in most studies. The gold standard for clinical trials of DPN has been nerve conduction velocity or subjective determination of nerve sensitivity. There is a need for objective endpoints encompassing earlier detection. Determination of sensory nerve density in the skin or cornea have been proposed to meet these criteria but clinical trials have not been performed to decide of their value (91). Another possibility that has not been given enough consideration is need for combination therapy. The etiology of DPN is complex and is impacted by dysfunction of numerous known and unknown pathways of metabolic and molecular disorders as well as structural damage. It is irrational to believe that monotherapy will be successful. For instance, hypertension is commonly treated with multiple drugs each directed at improving a causative event. The same approach needs to be considered for DPN.

If combination therapy is the best approach what evidence is there to suggest a specific combination of agents? We have extensively studied the effect of the combination of several different treatments in diabetic rodents focusing on neural and vascular endpoints. An important issue is that individually each of these agents have been safely used in humans and in combination would be predicted to have a good safety profile. Another consideration of the benefit of combination therapy is that precise dosing of each agent would not necessarily be needed to achieve the desired effect, thereby reducing the potential of unwanted side effects.

Our early studies demonstrated that vascular dysfunction of epineural arterioles that provide circulation to the sciatic nerve preceded slowing of nerve conduction velocity in streptozotocin-induced diabetes (20). This indicated a major role for vascular impairment in DPN. Vascular reactivity of these epineurial arterioles is mediated by different vasoactive compounds including acetylcholine, endothelial derived hyperpolarizing factor, calcitonin gene-related peptide (CGRP), bradykinin, insulin, and type C natriuretic peptide (CNP) (92, 93). Three of these peptides CGRP, bradykinin, and CNP are degraded by neutral endopeptidase (NEP) (94, 95). NEP activity increases with obesity and diabetes (96, 97). This stimulated our interest in the role NEP may have in regulating vascular function and DPN.

Studies in both types 1 and 2 diabetic rats have demonstrated that impaired vascular relaxation of the small blood vessels that provide circulation to the sciatic nerve occurs prior to the development of nerve dysfunction such as slowing of motor nerve conduction velocity (19, 20). In addition, our studies have indicated that increased oxidative and inflammatory stress are factors leading to impaired vascular function of these resistance vessels in diabetes (70, 98). These data have led us to focus our pre-clinical studies on interventions that reduce oxidative and inflammatory stress to improve vascular and nerve function.

## AVE7688 & LCZ7696

After a series of studies our interest turned to a combination therapy consisting of inhibition of angiotensin converting enzyme (ACE) and NEP or neprilysin. We and other had shown in animal models and human subjects that inhibition of ACE improved diabetic peripheral neuropathy (99, 100). In a clinical retrospective cohort study of patients with type 2 diabetes it was concluded that pharmacological inhibition of the angiotensin system is beneficial to prevent DPN (101). Our previous studies had also demonstrated that vascular relaxation by epineurial arterioles that provide blood flow to the sciatic nerve is mediated by CNP and CGRP (95, 102). CNP is expressed in the outer membrane of endothelial cells of epineurial arterioles, whereas CGRP is expressed in sensory nerves that innervate these blood vessels (95). Interestingly, NEP degrades natriuretic peptides such as CNP, CGRP, adrenomedullin, bradykinin, and endothelin (103). NEP expression is widespread and found in many tissues including vascular tissue, and in regard to diabetes, its activity is increased by fatty acids and glucose in human microvascular cells (104–108). We have discussed that diabetes leads to an increase in protein kinase C and in vascular tissue protein kinase C stimulates NEP activity (109, 110). We have shown that this increase of NEP expression and activity occurs in epineurial arterioles of the sciatic nerve of streptozotocin-treated rats (95). The first inhibitor combination of ACE and NEP we studied was AVE7688, a vasopeptidase inhibitor, later to be called Ilepatril. In those studies, we used both type 1 or type 2 diabetic rats and an intervention protocol to determine the efficacy of AVE7688 in DPN. AVE7688 reversed the diabetes-induced decrease in endoneurial blood flow, improved acetylcholine-and CGRP-mediated vascular relaxation by epineurial arterioles and reduced superoxide and nitrotyrosine levels in these resistance vessels, prevented the development of hypoalgesia in the hindpaw and significantly improved motor and sensory nerve conduction velocity (95, 111). These studies suggest that this class of drug may be an effective approach for the treatment of diabetic vascular and DPN. However, in studies with human subjects Ilepatril was found to increase the risk for angioedema and clinical development of this drug was stopped (112). As one door closes another open, and this led to the development of a new drug combination of angiotensin receptor blocker and neprilysin inhibitor. This new combination drug that highlights the use of angiotensin II receptor blocker rather than ACE inhibitor reduced the risk of angioedema (112). LCZ696 (ENTRESTO^®^) was the first representative of this class of drug and combines the dual action of sacubitril and valsartan (112). Sacubitril is a neprilysin/NEP inhibitor and valsartan is a well-known angiotensin II receptor antagonist. In a clinical trial sacubitril/valsartan was shown to be an effective treatment for heart failure with reduced ejection fraction and side effects comparable to the ACE inhibitor enalapril (113). Sacubitril/valsartan has obtained Food and Drug Administration (FDA) approval for treatment of heart related conditions but had not been tested for DPN. Because vasopeptidase inhibitors like AVE7688 and sacubitril/valsartan have similar targets we were interested in examining the efficacy of sacubitril/valsartan on diabetic vascular and DPN. A comprehensive study was designed using early and late intervention protocols. Treatment was initiated 4- or 12-weeks after the induction of hyperglycemia in a late-stage type 2 diabetic rat model. Treatment was given daily by gavage for 12 weeks followed by an extensive evaluation of vascular and neural endpoints (114). The highlights of the results were efficacy of sacubitril/valsartan treatment were superior in improving vascular and neural function than valsartan alone. In the early intervention protocol sacubitril/valsartan treatment slowed the progression of DPN impacting all relative endpoints, and intervention 12-weeks after induction of hyperglycemia stimulated restoration of vascular reactivity, motor and sensory nerve conduction velocities and sensitivity/regeneration of sensory nerves of the skin and cornea. We attributed the beneficial effects observed with sacubitril/valsartan treatment to reduction in oxidative stress and protection of neuro- and vaso-active peptides. These pre-clinical studies provide support for this class of drugs as a potential new treatment for DPN. Even though LCZ696 has FDA approval for another important clinical problem and has a good safety profile no further research has been done with this compound for DPN.

## Enalapril, α-lipoic acid & menhaden oil

Based upon individual results as mono-therapies we tested the combination of enalapril, α-lipoic acid and menhaden (fish) oil on peripheral neuropathy (115, 116). In a study with type 2 diabetic rats treatment consisting of enalapril, menhaden oil or α-lipoic acid as monotherapies or their combination were initiated 16 weeks after induction of hyperglycemia and treatment lasted 12 weeks (116). Prior to and after treatments analyses of an array of neural and vascular endpoints including cornea sensitivity, corneal nerve density, vascular reactivity of epineurial arterioles, motor and sensory nerve conduction velocity, intraepidermal nerve fiber density and thermal nociception were performed. Untreated diabetes caused an impairment of 20-40% in all endpoints. Vascular relaxation, nerve conduction velocity and thermal nociception worsened in untreated diabetic rats over the period of the study. The monotherapy treatment of diabetic rats was effective in improving neural and vascular deficits with menhaden oil being the most efficacious. Glucose clearance, a marker for insulin resistance, was impaired in untreated diabetic rats and significantly improved only with combination therapy. Diabetes caused steatosis, elevated serum lipid levels, slowed motor and sensory nerve conduction velocity, caused thermal hypoalgesia and reduction in intraepidermal nerve fiber density, decreased cornea sub-basal nerve fiber length and corneal sensitivity and caused impairment in vascular relaxation to acetylcholine and CGRP in epineurial arterioles of the sciatic nerve. Treating diabetic rats with the combination of enalapril, menhaden oil or α-lipoic acid served as a form of rehabilitation therapy, with nearly all the vascular and nerve deficits to revert to near control values with the lone exception of motor nerve conduction velocity, which was also significantly improved compared to diabetic rats but remained significantly decreased compared to control rats. As mono therapies each of these compounds/drugs have an excellent safety profile but have never been tested in humans as a combination. Each alone have been shown in humans to provide benefit to peripheral nerve dysfunction and other problems such as cardiovascular disease (100, 117, 118). It would be predicted that a combination of these compounds would be safe and have the potential to improve DPN but has not been tested in human subjects.

## Menhaden oil & salsalate

**Figure 4:**
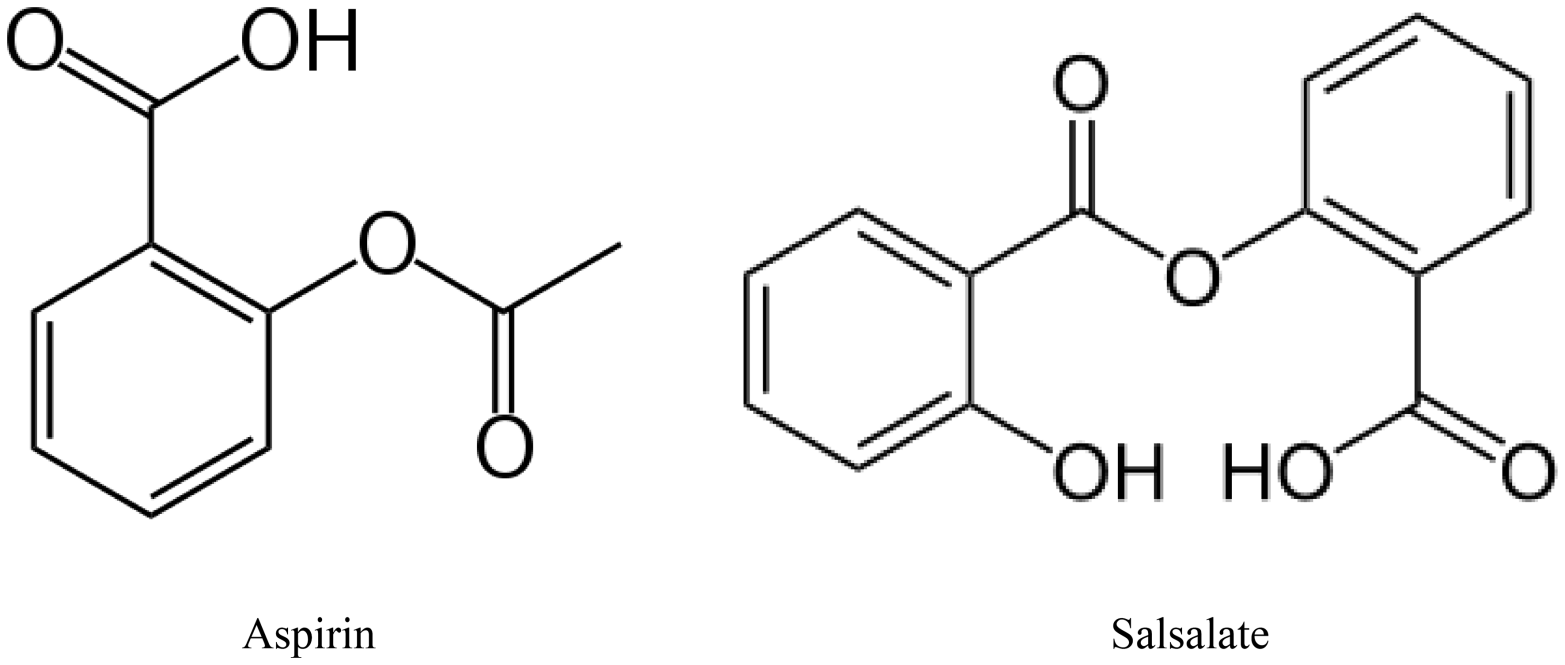
Metabolites of EPA and DHA.

**Figure 5:**
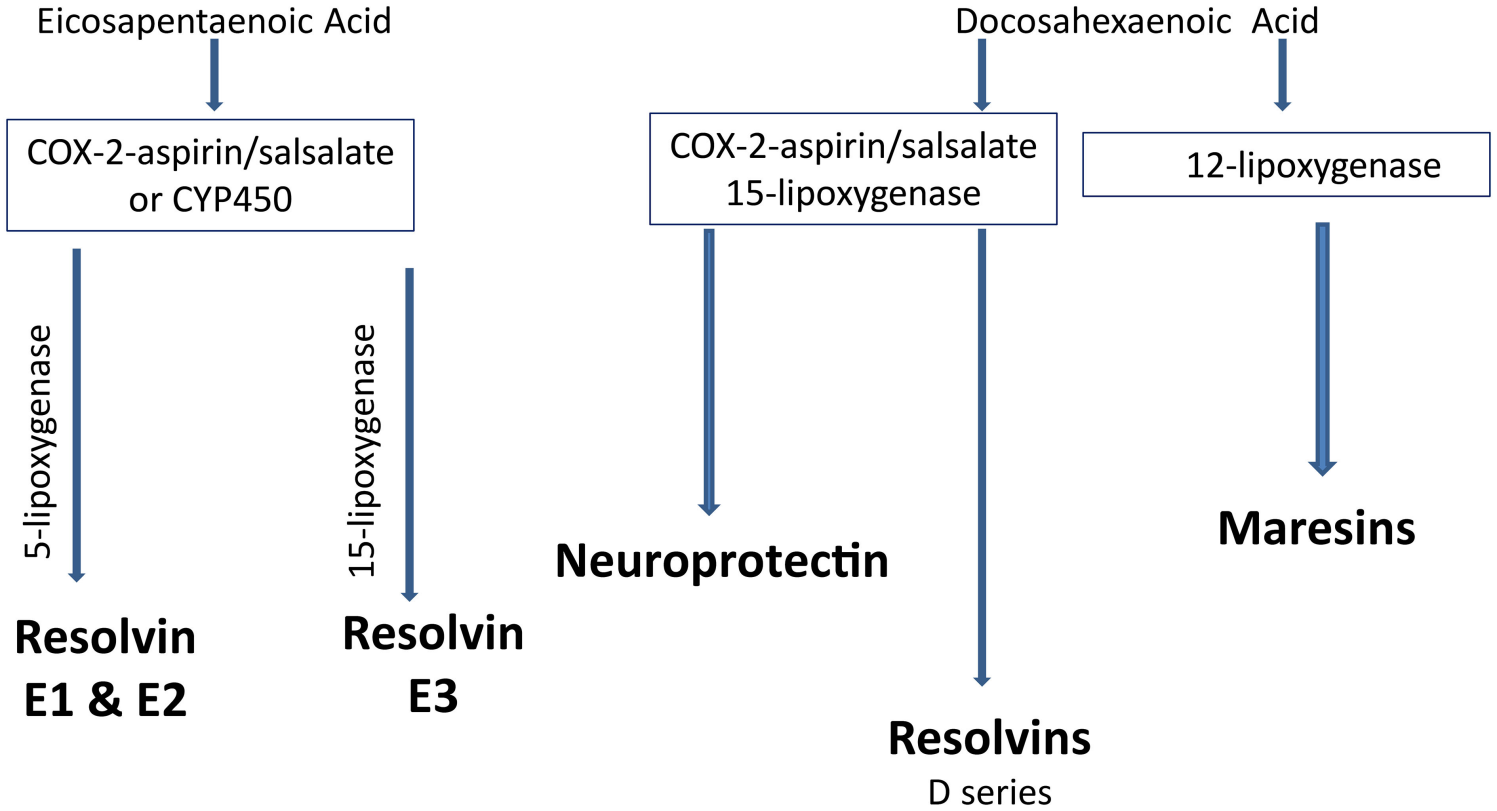
Metabolites of EPA and DHA.

Increasing consumption of omega-3 (n-3) polyunsaturated fatty acids, commonly found in marine mammals, (eicosapentaenoic acid (EPA) and docosahexaenoic acid (DHA) through the diet or by supplements has been shown to improve cardiovascular disease (117, 119). Increased inflammatory stress is associated with a poor n-6 to n-3 ratio of polyunsaturated fatty acids in circulation (120, 121). Metabolites of EPA, E series resolvins and DHA, neuroprotectin D1, D series resolvins and maresins have been shown to have anti-inflammatory and neuroprotective properties (122–124). Salicylsalicylic acid (salsalate), a non-acetylated salicylate, is a nonsteroidal anti-inflammatory agent that inhibits the synthesis of prostaglandins by inactivating cyclooxygenase enzymes (**Figure 4**) (125). Salsalate, like aspirin, in combination with fish oil increases the production of resolvins from EPA and DHA (**Figure 5**) (126). Salsalate is insoluble in the stomach and moves to the small intestine where it is hydrolyzed into two salicylic acid molecules. Treatment of human subjects with type 2 diabetes with salsalate has been shown to improve insulin resistance (127, 128). This led us to examine the combination of omega-3 polyunsaturated fatty acids (fish oil) and salsalate on DPN. We hypothesized that the addition of salsalate to omega-3 polyunsaturated fatty acids would increase the formation of omega-3 polyunsaturated fatty acid metabolites including E and D series resolvins that we have previously shown to be as effective as fish oil alone in improving DPN in type 2 diabetic mice (126). In a study with type 2 diabetic rats, we verified our hypothesis by showing that adding salsalate to high fat diets enriched with 10% or 25% kcal of menhaden (fish) oil prevented the diabetes-induced decrease in motor and sensory nerve conduction velocity, intraepidermal nerve fiber density thermal nociception, cornea nerve density and sensitivity as well as vascular relaxation to acetylcholine and CGRP, to a greater extent than 10% or 25% menhaden oil alone (129). Our study also showed that the combination of salsalate with menhaden oil increased the circulating levels of resolvin D1 significantly compared to menhaden oil alone. When using a higher dose of menhaden oil alone (45% kcal) we found that the vascular and neural functions were maximally protected and adding salsalate did not provide any additional benefit. In addition, the use of salsalate alone in the high fat diet of type 2 diabetic rats provided only minimal protection/improvement of vascular and neural dysfunction. The primary message from these studies was that the effects of dietary salsalate in combination with lower amounts of menhaden oil are at minimum additive and perhaps synergistic toward diabetes-induced vascular and peripheral neuropathy endpoints.

## Summary

DPN is a disease with multiple etiologies thereby providing many potential targets for treatment. Even though a mono-therapeutic approach has provided success in rodent studies successful translation to humans has not occurred. There is a movement toward combination therapy to alleviate pain for painful diabetic neuropathy (130, 131). Therefore, it is difficult to understand the reluctance to take the same approach to provide a disease modifying therapy for DPN. Above I have summarized several combinations that my laboratory has used to treat DPN in rodent models. The literature provides more examples that were not reviewed here. The encouraging fact that for each drug or compound in these combinations have been shown to be safe for humans. Future clinical trials should consider using updated primary endpoints that do not take years to develop such as such as nerve conduction deficits and a combination of drugs/compounds that are safe for human use combined with proper lifestyle modifications in diet and exercise (132).

If asked what a successful combination treatment for DPN would look like I would base my answer on the pre-clinical studies we have performed. I would consider a combination of angiotensin converting enzyme inhibitor or angiotensin receptor blocker, omega-3 polyunsaturated fatty acid supplement of 2 – 3 g per day, aldose reductase inhibitor and α-lipoic acid. Each of these compounds have been used by human subjects with a high degree of safety, thereby reducing the likelihood of unknown side effects. However, when combining drugs there is always a risk of unknown or additional side effects and this would need to be monitored with treatment. In addition, to these treatments improving diet and exercise should be recommended using the guidelines provided by the American Diabetes Association. When discussing weight loss consideration of treatment with a glucagon-like peptide-1 receptor agonist needs to be acknowledged. This class of drugs are not new but have gained attention recently as an effective treatment for obesity and type 2 diabetes (133–135). There is also evidence that these drugs are effective for DPN (136, 137). There are few pre-clinical studies available about the interaction of glucagon-like peptide-1 receptor agonists with the other drugs discussed above and if included in combination with other drugs monitoring of unknown side effects would be needed.

In conclusion, to find a successful treatment for DPN there is a need for standardized diagnostic criteria for early detection, reliable biomarkers, and innovative combination treatment approaches as discussed above (138).

## Author contributions

MY: Conceptualization, Funding acquisition, Investigation, Methodology, Project administration, Resources, Validation, Visualization, Writing – original draft, Writing – review & editing.
